# Effects of the Natural Flavonoid Quercetin on Arenavirus Junín Infection

**DOI:** 10.3390/v15081741

**Published:** 2023-08-15

**Authors:** Aaron Ezequiel Alvarez De Lauro, Miguel Angel Pelaez, Agostina Belén Marquez, Mariel Selene Wagner, Luis Alberto Scolaro, Cybele Carina García, Elsa Beatriz Damonte, Claudia Soledad Sepúlveda

**Affiliations:** Laboratory of Antiviral Strategies, Biochemistry Department, School of Sciences, University of Buenos Aires, IQUIBICEN, University of Buenos Aires/Consejo Nacional de Investigaciones Científicas y Técnicas, Buenos Aires 1428, Argentina

**Keywords:** arenavirus, Junín virus, Argentine hemorrhagic fever, quercetin, natural flavonoid, pretreatment, antiviral cell state, virus entry, PI3K/Akt pathway, transferrin receptor

## Abstract

There is no specific chemotherapy approved for the treatment of pathogenic arenaviruses that cause severe hemorrhagic fever (HF) in the population of endemic regions in America and Africa. The present study reports the effects of the natural flavonoid quercetin (QUER) on the infection of A549 and Vero cells with Junín virus (JUNV), agent of the Argentine HF. By infectivity assays, a very effective dose-dependent reduction of JUNV multiplication was shown by cell pretreatment at 2–6 h prior to the infection at non-cytotoxic concentrations, with 50% effective concentration values in the range of 6.1–7.5 µg/mL. QUER was also active by post-infection treatment but with minor efficacy. Mechanistic studies indicated that QUER mainly affected the early steps of virus adsorption and internalization in the multiplication cycle of JUNV. Treatment with QUER blocked the phosphorylation of Akt without changes in the total protein expression, detected by Western blot, and the consequent perturbation of the PI3K/Akt pathway was also associated with the fluorescence redistribution from membrane to cytoplasm of TfR1, the cell receptor recognized by JUNV. Then, it appears that the cellular antiviral state, induced by QUER treatment, leads to the prevention of JUNV entry into the cell.

## 1. Introduction

The *Arenaviridae* family includes the genus *Mammarenavirus*. These arenaviruses infect mammals and represent a serious and growing challenge for human public health. In particular, Lassa virus (LASV) and Junín virus (JUNV) cause periodic annual outbreaks of severe human hemorrhagic fever (HF) in endemic areas of West Africa and Argentina, respectively [1,2]. However, there is still no safe and effective chemotherapy against these infections. The treatment is limited to the use of ribavirin, effective against Lassa HF, by intravenous administration [3], carrying important associated adverse effects and limitations, or to the administration of convalescent plasma with defined doses of JUNV neutralizing antibodies, recommended for patients with Argentine HF [4]. On this basis, there is an essential need for the development of other therapeutic options against arenaviruses, employing viral target-based approaches aimed to block a virus-encoded function or host-targeted approaches that inhibit any cellular function required for viral multiplication [1].

Natural compounds have gained popularity in recent decades due to their diverse biological functions and their direct application as treatments [5]. Polyphenols are naturally occurring chemical compounds present in plants, divided into two main groups: flavonoids and non-flavonoids. Quercetin (QUER), the major representative of the flavonoid subclass of flavonols [5], is a secondary metabolite that possesses multiple pharmacological properties such as antioxidant, anticancer, antiaging, anti-inflammatory and antiviral [5,6,7,8,9,10,11,12,13,14]. QUER is capable of inhibiting cell proliferation, inducing apoptosis and auto-phagy, inhibiting metabolic process like adipogenesis and lipogenesis [15,16,17], and regulating different signaling pathways such as Wnt/3-catenin, MAPK/ERK, MAPK/JNK, PI3K/AKT/mTOR, MAPK/p38, p-53 and NF-kB [18]. 

Recently, many mechanisms of the antiviral action of QUER were described against several RNA and DNA viruses, including severe acute respiratory syndrome coronavirus 2 [5,8,13,19,20], and other ones were proposed through the integration of in silico approaches [13,21]. Therefore, in this study, we evaluated the antiviral activity of QUER on JUNV multiplication in different cell culture systems at different stages of the viral replication cycle, as well as the direct virucidal activity. 

## 2. Materials and Methods

### 2.1. Reagents

Quercetin (QUER) [2-(3,4-Dihydroxyphenyl)-3,5,7-trihydroxy-4H-1-benzopyran-4-one, 3,3′,4′,5,6-Pentahydroxyflavone] was purchased from Sigma-Aldrich (St. Louis, MO, USA). Compound stock solution was prepared in dimethyl sulfoxide (DMSO) ReagentPlus^®^, ≥99.5% (Sigma-Aldrich, St. Louis, MO, USA) at 100 mg/mL.

### 2.2. Cell Culture

Human lung carcinoma A549 cells (ATCC CCL-185) and African green monkey Vero cells (ATCC CCL-81) were cultured in minimum essential medium Eagle (MEM, Gibco™ Thermo Fisher Scientific, Waltham, MA, USA) supplemented with 5% heat-inactivated newborn bovine serum (NBS, Gibco™ Thermo Fisher Scientific, Carlsbad, CA, USA), and 1.5 g/L of sodium bicarbonate in a 5% CO_2_ atmosphere. For the maintenance medium (MM), the serum concentration was reduced to 1.5%.

### 2.3. Viruses

The experiments were performed using the naturally attenuated IV4454 strain of JUNV obtained from a mild human case of Argentine HF [22], Tacaribe virus TRLV 11573 strain (TCRV) and lymphocytic choriomeningitis virus WE strain (LCMV). Virus stocks were propagated in Vero cells and titrated by plaque forming unit (PFU) quantification in the same cell line.

### 2.4. Viral Titrations

Viral titers were determined by standard plaque assay. Supernatants were collected and serial dilutions were prepared to infect Vero cell monolayers for 1 h at 37 °C. After viral adsorption, the excess virus was removed; MM supplemented with 0.7% methylcellulose was added and cells were incubated for 1 week at 37 °C in a 5% CO_2_ incubator. Finally, cell monolayers were fixed with 10% formaldehyde, washed, stained with a 1% solution of crystal violet and PFU were counted. 

### 2.5. Cytotoxicity and Anti-Proliferative Assay

Cell viability was measured in resting and actively growing cells. For resting cells, confluent cell monolayers in 96-well plates (5 × 10^4^ cells per well) were exposed for 48 h to serial two-fold compound dilutions, three wells for each concentration, and then viability was tested. To monitor the proliferation of actively growing cells, 1 × 10^4^ cells were seeded in 96-well plates and allowed to adhere during a 2.5 h incubation period at 37 °C. Thereafter, compound was added, followed by a 48 h incubation. Cell viability determinations were performed by the 3-(4,5-dimethylthiazol-2-yl)-2,5-diphenyl tetrazolium bromide (MTT) (Sigma-Aldrich, St. Louis, MO, USA) method, for which 10 µL of MM containing MTT reagent (final concentration 0.5 mg/mL) was added to each well. After 2 h of incubation at 37 °C, the supernatants were removed, ethanol was added to each well and absorbance was measured at 595 nm [23]. The cytotoxic concentration 50% (CC_50_) was calculated by linear regression using GraphPad Prism (v8.0.1) software, as the compound concentration was required to reduce the MTT signal by 50% compared to cell controls treated with vehicle DMSO. The anti-proliferative effect of QUER was also determined by the colony forming cell (CFC) assay. For this, cells were treated as detailed above and fixed after 48 h with 10% formaldehyde, stained with a 0.05% crystal violet in ethanol solution and washed thoroughly. Then, cell colonies were visualized and images were captured using an optical microscope (Nikon Eclipse TS100) at a magnification of 40×. Finally, stained colonies were quantified by solubilizing crystal violet in 50% ethanol, 0.1% acetic acid, and measuring the solution absorbance at 595 nm.

### 2.6. Antiviral Activity

The antiviral activity was determined under different conditions. Pretreatment: cells were incubated with different two-fold dilutions of QUER in MM for 2, 6 or 24 h. Then, cells were washed three times with phosphate-buffered saline (PBS) (Gibco™ PBS, pH 7.4 cat# 10010023), infected at 37 °C with each virus at a multiplicity of infection (m.o.i.) of 0.1, washed three times with PBS and refed with compound-free MM. Compound after adsorption: cells were infected in the absence of compound, and after 1 h adsorption at 37 °C the unadsorbed virus was removed by washing with PBS, and refed with MM containing different two-fold dilutions of QUER. For both treatments, extracellular virus yields were determined after 48 h of infection by plaque formation. The effective concentration 50% (EC_50_) was calculated as the concentration required to reduce virus yield by 50% in the compound treated cultures, compared with untreated ones by linear regression using GraphPad Prism (v8.0.1) software.

### 2.7. Virucidal Assay

The virucidal effect of QUER was determined mixing up aliquots of a viral suspension containing approximately 1 × 10^6^ PFU with the same volume of the appropriate QUER working solution, followed by the incubation at the indicated temperature. In parallel, as a control, an equivalent aliquot of the virus suspension was incubated with MM plus DMSO (dilutions of solvent corresponding to the working solutions of QUER) under the same conditions. Then, samples were cooled down, diluted with MM and used to determine the remaining infectivity by plaque assay method. The concentration required to inactivate virions by 50% (IC_50_) was then calculated from the dose–response inactivation curve.

### 2.8. Time of Drug Addition Assay

Cells grown in 24-well microplates were infected with JUNV (m.o.i. 2) for 1 h at 4 °C in MM containing 50 µg/mL of QUER (time 0) or MM without compound. After removing the inocula, cells were washed with PBS; MM with 50 µg/mL of QUER was added immediately (time 0 and 1 h p.i.) or at different times p.i. (2 to 12 h p.i.) and incubation was followed at 37 °C. An infected culture without drug treatment was performed simultaneously as a control. In all cases, extracellular virus yields were determined by plaque formation at 24 h p.i.

### 2.9. Viral Entry

Cells were infected with JUNV and treated with 50 µg/mL of QUER under different conditions. Compound during adsorption: cells were infected with MM containing, or not, 50 µg/mL of QUER; after 1 h adsorption at 4 °C, inocula were discarded, cells were washed three times with PBS and refed with compound-free MM. Compound during internalization: cells were infected with MM; after 1 h adsorption at 4 °C, inocula were discarded, cells were washed three times with PBS to remove the unadsorbed virus and refed with MM containing, or not, 50 µg/mL of QUER at 37 °C. After 2 h, cells were washed three times with PBS and refed with compound-free MM. In all cases, extracellular virus yields were determined by plaque formation at 48 h p.i.

### 2.10. Western Blot (WB)

Cells grown in 24-well microplates were treated, or not, with QUER 50 µg/mL for 2 h. Then, cells were washed with PBS and lysed with 50 µL of equal parts of MilliQ water added with 10% β-mercaptoethanol and 2× Laemmli sample buffer (Bio-Rad, Hercules, CA, USA). Proteins from whole-cell lysates were separated by 10% SDS-PAGE (Bio-Rad, Hercules, CA USA), and electrophoresis was performed at 150 V in running buffer (Bio-Rad, Hercules, CA, USA). Protein transfer was performed on a PVDF transfer membrane (Thermo Fisher Scientific, Waltham, MA, USA), using a liquid electrotransfer system (Mini Trans-Blot^®^ Bio-Rad, Hercules, CA, USA) for 2 h at 0.7 mA/cm^2^ of membrane, previously activated with methanol and equilibrated with transfer buffer (running buffer without SDS, 20% methanol). After transfer, the membranes were blocked and revealed by using anti-phospho-Akt (Ser473) (1:500) (CST 9271, New England Biolabs, Ipswich, MA USA) and anti-transferrin receptor 1 (TfR1) antibodies (1:250) (anti-human CD71, BD Pharmigen^TM^, BDIS, San Jose, CA, USA) as previously described [24]. Membranes were stripped and re-probed with anti-Akt (CST 9272, New England Biolabs, Ipswich, MA USA) and anti-β-Actin antibodies (1:2000) (A5441 Sigma-Aldrich, St. Louis, MO, USA) to ensure equal protein loading. Chemiluminescence detection was performed using Biolumina (Kalium Techmologies, Buenos Aires, Argentina) and protein bands were quantified by FIJI software (National Institutes of Health, Bethesda, MD, USA). 

### 2.11. Indirect Immunofluorescence

Cells grown over glass coverslips were treated with MM containing, or not, 50 µg/mL of QUER. After 2 h, cells were washed with PBS and processed to immunofluorescence assay. Cells were fixed by incubation in 4% paraformaldehyde for 15 min at room temperature, washed three times with PBS and finally permeabilized by incubation with 0.1% Triton X-100 in PBS for 15 min at room temperature. Then, cells were rinsed three times with PBS before incubation with anti-TfR1(1:250) (anti-human CD71, BD PharmigenTM, BDIS, San Jose, CA USA) for 1 h at 37 °C and then incubated at 37 °C with anti-mouse Alexa 488 (1:300) (A-11001, ThermoFisher Scientific, Waltham, MA, USA) conjugated antibodies. Cell nuclei were stained using 4′,6-diamidino-2-phenylindole (DAPI) (1:1000) (Sigma-Aldrich, St. Louis, MO, USA). Coverslips were mounted in a glycerol solution containing 1,4-diazabicyclo [2,2,2]octane (DABCO). Then, cells were visualized and images were captured using the confocal laser scanning microscope ZEISS LSM 980 at a magnification of 63×. The fluorescence intensity of TfR1 present in plasma membrane and cytoplasm was quantified. For that, the immunofluorescence assay was carried out as aforementioned, and stained with Wheat Germ Agglutinin (WGA) conjugated with tetramethyl rhodamine isothiocyanate (TRITC) (1:200) (Sigma-Aldrich Co., St. Louis, MO, USA). Cells were then visualized using the epifluorescence microscope Olympus IX71, magnification 60×. The analysis of the data was performed with the free software Image J, counting 30 cells per sample. For the quantification of TfR1 present in the plasma membrane, an unsharp mask was generated over the WGA-labelled cells image (red) using the MaxEntropy algorithm. Shaded regions were selected and then dragged into the original image labelled with anti-TfR1 (green). Finally, the integrated density (IntenDen), the mean and cell areas were measured. TfR1 present in cell cytoplasm was measured by shading cell images manually in order to select the cell cytoplasm areas. Shaded regions were selected and dragged into the original images labelled with anti-TfR1 (green); thereafter, the IntenDen, the mean and cell areas were measured. Additionally, the background mean and the mean of cell areas were measured in order to quantify the fluorescence intensity, correcting these parameters. Values were presented as the corrected total cell fluorescence (CTCF) = IntenDen − (Area of selected cell × Mean fluorescence of background readings).

### 2.12. Statistical Analysis

Statistical analysis was performed using GraphPad Prism (v8.0.1) software. Comparison of means was tested by one-way or two-way analysis of variance (ANOVA) with Dunnett’s post hoc test or utilizing the t-student test. Statistical significance was defined as *p* < 0.05 (95% confidence interval). * *p* < 0.05; ** *p* < 0.001; *** *p* < 0.0001; **** *p* < 0.00001.

## 3. Results

### 3.1. Effect of QUER on Cell Viability and Proliferation

**Figure 1:**
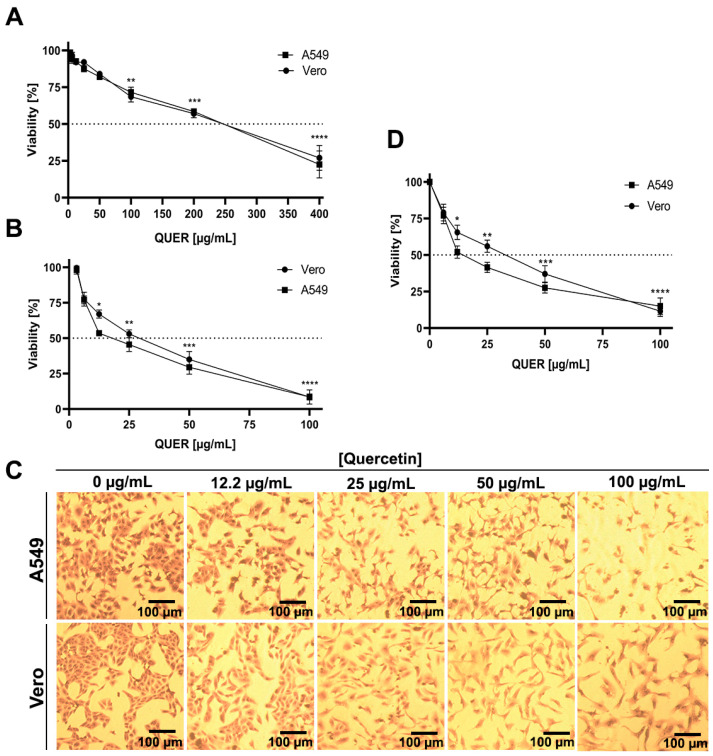
Cytotoxicity and anti-proliferative effect of QUER. To measure the effects on (**A**) confluent or (**B**) actively growing A549 or Vero cells, two-fold dilutions of QUER were added 24 or 2.5 h after cell seeding in 96-well plates, respectively. After 48 h of incubation, the ratio of viable cells in drug-treated and mock-treated cultures was determined by MTT assay and expressed as % cell viability. (**C**) Colony forming cell assay (CFC): Actively growing A549 (upper panel) and Vero (lower panel) cells were treated with QUER as in (**B**), after 48 h at 37 °C cells were fixed and stained with 1% crystal violet solution. Images were obtained using optical microscope Nikon Eclipse TS100. Magnification 40×. (**D**) Stained cell colonies were quantified by absorbance of solubilized crystal violet. Viability values (% respect to non-treated cell control) are presented as the mean ± SD obtained from three independent experiments. * *p* < 0.05; ** *p* < 0.001; *** *p* < 0.0001; **** *p* < 0.00001.

The effects of QUER treatment on host cells were analyzed by monitoring the viability of confluent cell monolayers and the proliferation of actively growing cells in the presence of different concentrations of the compound. When QUER cytotoxicity was determined by the MTT method in uninfected confluent cultures of A549 and Vero cells after 48 h of treatment, no significant reduction of cell viability was detected up to 50 µg/mL of QUER (Figure 1A). The CC_50_ values were 224.8 ± 1.2 µg/mL and 229.5 ± 1.0 µg/mL for A549 and Vero cells, respectively. However, when QUER was added 2.5 h after seeding the cells, the increase of cell number after 48 h of exposure to the compound was significantly altered at lower concentrations in both cell lines, compared to the untreated cell control (Figure 1B), an effect that was also seen in the size of cell colonies in a concentration-dependent manner, determined by crystal violet staining (Figure 1C) and quantified by absorbance (Figure 1D).

### 3.2. Effect of QUER on JUNV Multiplication

**Figure 2:**
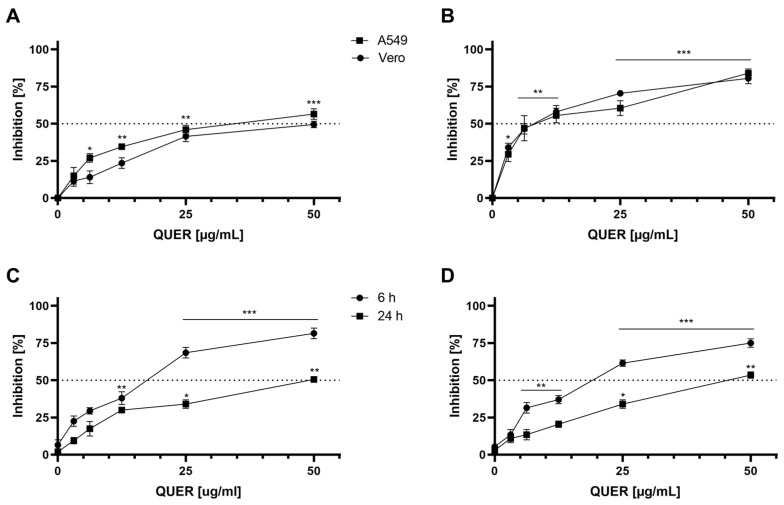
Antiviral activity of QUER against JUNV. The antiviral activity of the compound was determined after 48 h of infection (m.o.i. 0.1) by plaque formation. (**A**) Cells were infected in the absence of compound and, after 1 h adsorption at 37 °C, refed with MM containing different dilutions of QUER. (**B**) After incubation with different dilutions of QUER in MM for 2 h at 37 °C, cells were infected with JUNV and refed with compound-free MM. (**C**) A549 or (**D**) Vero cells were treated as in (**B**) for 6 or 24 h before viral infection. Inhibition values (% respect to non-treated viral control) are presented as the mean ± SD obtained from three independent experiments. * *p* < 0.05; ** *p* < 0.001; *** *p* < 0.0001.

In order to study the impact of the compound on the JUNV replication cycle, extracellular production of viral particles was assessed 48 h after treatment with different non-cytotoxic concentrations of QUER. A dose-dependent inhibition of viral yields was observed when the compound was added after viral adsorption, reaching up to 60% inhibition with the maximum concentration evaluated (Figure 2A). The EC_50_s for this treatment were 32.48 ± 1.1 µg/mL for A549 and 46.76 ± 4.3 µg/mL for Vero cells. On the other hand, the 2 h treatment of cell monolayers prior to JUNV infection produced inhibition levels higher than 85% using the same range of concentrations. In this case, the EC_50_s resulted in 6.1 ± 0.7 µg/mL for A549 and 7.5 ± 2.5 µg/mL for Vero cells (Figure 2B).

Based on these results, the impact of QUER over viral yields prior to viral infection was further studied. To this end, the cell monolayers were pretreated with the same range of concentrations as in the previous assays during 6 and 24 h, then cells were infected and incubated at 37 °C for 48 h in the absence of QUER. As shown in Figure 2C,D, high levels of viral inhibition were obtained by pretreating cells with QUER for 6 h, but significantly decreased in cultures treated for 24 h prior to the viral infection in both cell lines.

**Figure 3:**
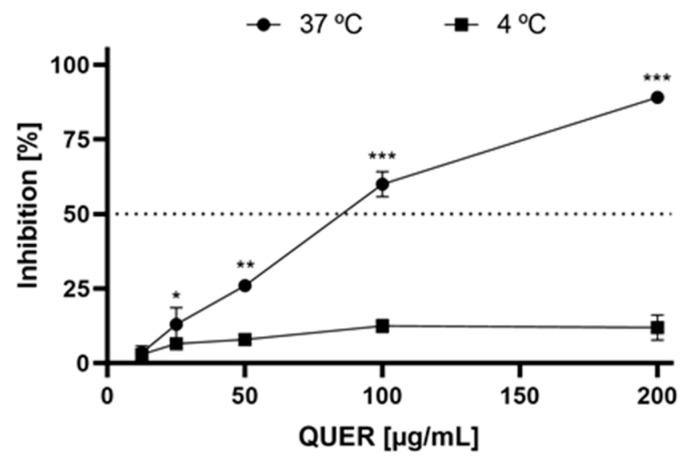
Virucidal effect of QUER. The inactivating effect of the compound was determined by plaque formation assay in Vero cells, after 1.5 h exposure (37 or 4 °C) to MM containing different dilutions of QUER. The remaining infectivity was determined by PFU. Inhibition values (% respect to non-treated viral control) are presented as the mean ± SD obtained from three independent experiments. * *p* < 0.05; ** *p* < 0.001; *** *p* < 0.0001.

The possibility of a virucidal activity of QUER was also analyzed as an initial point to understand the inhibitory effect of this compound against JUNV. When QUER was applied directly to JUNV particles, the infectivity was reduced, suggesting that this compound affects the virion integrity. This virucidal effect was evident at 37 °C (IC_50_ = 81.7 ± 4.3 µg/mL) but was not detected when incubation was carried out at a lower temperature (IC_50_ > 200 µg/ mL) (Figure 3).

### 3.3. Mechanism of Antiviral Action of QUER on JUNV

**Figure 4:**
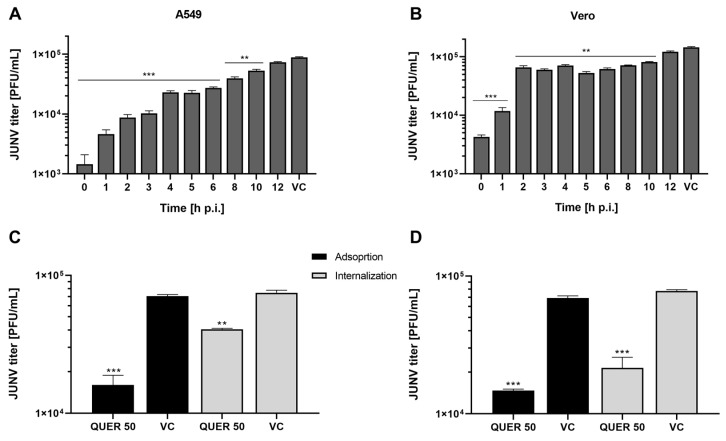
Effect of time of treatment with QUER on antiviral activity. (**A**) A549 or (**B**) Vero cells were infected with JUNV (m.o.i 2) in the presence (0 h p.i.) or absence of compound (1 to 12 h p.i.). After 1 h at 4 °C, cell cultures were refed with MM and incubated at 37 °C. At different times p.i. cells were treated with 50 µg/mL of QUER. Extracellular virus yields were determined by plaque formation at 24 h p.i. (**C**) A549 or (**D**) Vero cells were infected with JUNV (m.o.i 2) under different treatment conditions. Adsorption: cells were infected in MM containing or not 50 µg/mL of QUER, after 1 h at 4 °C, cells were refed with compound-free MM. Internalization: virus was adsorbed to cells for 1 h at 4 °C in MM, then MM with or without 50 µg/mL of QUER was added, after 2 h incubation at 37 °C supernatant was removed and cells were covered with compound-free MM. For both treatments, extracellular virus yields were determined after 48 h p.i. by PFU. Virus titers are presented as the mean ± SD obtained from three independent experiments. ** *p* < 0.001; *** *p* < 0.0001.

In order to elucidate the mechanism of antiviral action of QUER against JUNV, a time of drug addition assay was performed. For this, the infection of cell cultures was synchronized by carrying out the viral adsorption at 4 °C and the compound was added at differ-rent times p.i. As shown in Figure 4A, QUER was much more effective to reduce JUNV multiplication when present during early times of infection, losing partially its effect when added after 3 h p.i., mostly in infected A549 cell cultures (Figure 4A). From these results, the inhibition of viral entry was studied in detail, discriminating the processes of viral attachment and viral entry into cells. Although both stages were affected by the presence of QUER, the effect was significantly lower than that obtained when cells were treated for 2 h prior to the infection with JUNV (Figure 4B; Figure 2B–D). Then, it appears that the cellular antiviral state induced by QUER leads to the prevention of viral entry.

In order to better understand how QUER would be affecting the multiplication of JUNV, the effect of the compound on the infection with other arenaviruses was compared. Assays were performed with TCRV, the prototype species of New World (NW) arenaviruses, which is closely related to JUNV, and LCMV, the prototype species of Old World (OW) arenaviruses, non-related to JUNV.

**Figure 5:**
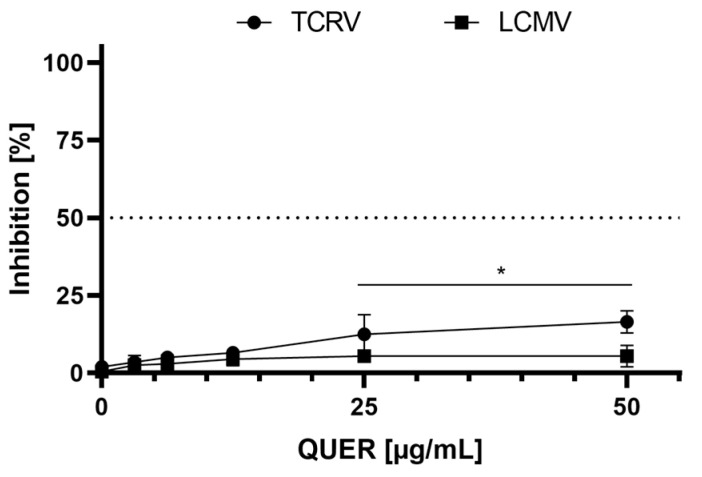
Antiviral activity of QUER against TCRV (•) and LCMV (▪). Vero cells were incubated with different dilutions of QUER in MM for 2 h at 37 °C, then washed, infected, unadsorbed virus removed and refed with compound-free MM. Viral yields were determined at 48 h p.i. and inhibition values (% respect to non-treated viral control) are presented as the mean ± SD obtained from three independent experiments. * *p* < 0.05.

As shown in Figure 5, QUER was able to inhibit the replication of TCRV in Vero cells when the cell cultures were only pre-treated for 2 h with QUER, but with a very low efficiency in comparison to JUNV, since under these conditions, the viral yield reduction was lower than 20% at all tested concentrations. In addition, when the same treatment was applied to culture infected with LCMV, QUER was completely inactive. Furthermore, a virucidal assay by direct contact of QUER with TCRV and LCMV particles did not reduce the remaining infectivity in either of the two cases, suggesting that this compound would not affect the integrity of the virions of these two arenaviruses. 

Results presented in Figure 2, Figure 4 and Figure 5 allow us to conclude that QUER exerts a differential effect against the in vitro multiplication of the mammarenavirus JUNV in comparison to other members of the same genus, such as TCRV and LCMV, suggesting that QUER may affect a cellular component involved in the initial stage of JUNV entry into the host cell.

The entry process is initiated when the envelope protein GP1 binds to the corresponding cellular receptor, and the OW and NW mammarenaviruses use different pathways. OW viruses, such as LASV and LCMV, as well as NW Clade C viruses, interact with the α-dystroglycan, an extracellular matrix protein commonly found in the basolateral membrane [25,26]. On the other hand, pathogenic NW clade B arenaviruses like JUNV interact with the human transferrin receptor 1 (TfR1) to enter into the cell [27]. In contrast, non-pathogenic viruses in this clade, such as TCRV, Amapari (AMAV) and Cupixi (CUPV) viruses, do not bind to human TfR1 [28]. Therefore, we decided to analyze the possible involvement of TfR1 on the inhibitory activity of QUER against JUNV infection.

In addition, it was shown that QUER is a phosphatidylinositol 3-kinase (PI3K) inhibitor [29,30]. The PI3K/Akt pathway is responsible for iron homoeostasis because it is directly involved in the transferrin (Tf) and TfRs recycling pathway. We previously demonstrated that JUNV infection is able to activate PI3K/Akt/protein kinase B (PKB) signaling pathway at an early stage of infection, and this activation, mediated by virus internalization, would be necessary for an efficient viral multiplication, particularly virus adsorption to cells mediated by TfR1 [24].

**Figure 6:**
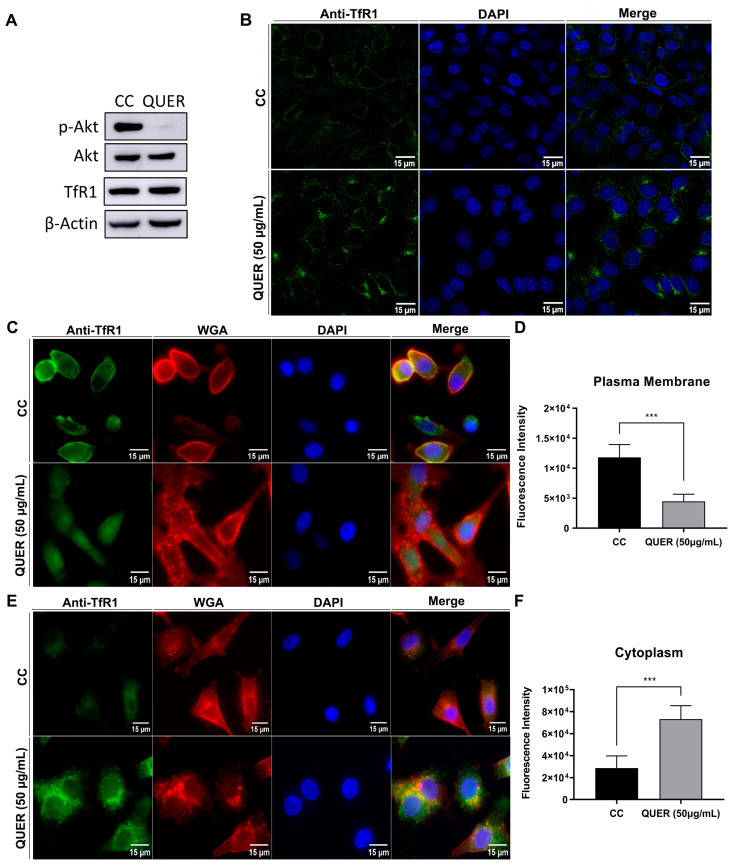
Effect of QUER on Akt and TfR1. (**A**) A549 cells were treated (QUER) or not (CC: cell control) with QUER 50 µg/mL during 2 h. Then, cells were lysated and proteins were separated, transferred onto a PVDF membrane, blocked and revealed by using anti-phospho-Akt, anti-Akt, anti-TfR1 and anti-β-actin antibodies. Chemiluminescence detection was performed and protein bands were quantified. (**B**) A549 cells were treated with (QUER) or without (CC) QUER 50 µg/mL during 2 h and then were processed to immunofluoescence assay. Cells were fixed, permeabilized, incubated with anti-TfR1 and then incubated with anti-mouse Alexa 488 conjugated antibodies. Cell nuclei were stained using DAPI. Coverslips were mounted, cells were visualized and images were captured by confocal microscopy (63×). (**C**,**E**) A549 cells were treated with (QUER) or without (CC) QUER 50 µg/mL during 2 h and then were processed to immunofluorescence assay without permeabilization (C) or permeabilized with Triton X-100 (**E**). Cells were stained for TfR1 as in B, and cell membrane was labelled using WGA-TRITC. Cells were then visualized using the epifluorescence microscope Olympus IX71, magnification 60×. (**D**,**F**) The analysis of the data was performed using the free software ImageJ, and statistical analysis was performed counting 30 cells per treatment using the t-student test. *** *p* < 0.0001.

With all of this in mind, the effect of QUER on Akt and TfR1 was next analyzed. A549 cells were treated for 2 h with 50 µg/mL of QUER and processed for WB to determine the degree of phosphorylated Akt or TfR1 expression level. As can be seen in Figure 6A, QUER treatment inhibited Akt phosphorylation without affecting Akt or TfR1 expression level, suggesting that this inhibition would be mediated by the inhibition of PI3K. Although expression of TfR1 is low in most normal cells, it is overexpressed (~100-fold) in many tumor cells, such as the A549 cell line, due to the increased iron demand [31]. For this reason, and taking into account the close relationship between the PI3K/Akt pathway and TfR1 recycling, we decided to analyze, by confocal microscopy, the possible involvement of TfR1 in the inhibitory activity of QUER against JUNV infection. The inhibition of Akt phosphorylation was reflected in a change in the cellular distribution of TfR1 fluorescence, which varied from a discrete punctate pattern, mostly located in the cell membrane to a dotted cytoplasmic one (Figure 6B).

To study the impact of QUER on TfR1 distribution, treatments were performed on uninfected A549 cells with 50 μg/mL of QUER for 2 h. Afterwards, cells were fixed under different conditions: treated with PFA in order to preserve cellular plasmatic membranes (PM) (Figure 6C) or permeabilized to explore all cellular membranes (PM/endoplasmic reticulum and Golgi membranes) (Figure 6E). Afterwards, samples were first stained for TfR1 and then labelled with WGA, a lectin that binds non-enzymatically to N-acetyl-D-glucosamine and sialic acid residues of glycoproteins and glycolipids present in the cellular membranes and observed in detail by microscopy. Images were analyzed and fluorescence intensity quantified, revealing that QUER-treated samples exhibited a decrease in fluorescence intensity in PM, and an increase in fluorescence intensity in cytoplasm (Figure 6D,F). Altogether, these results suggest that the presence of the viral receptor may be affected and involved in the mechanism of antiviral action of QUER against JUNV in A549 cells.

## 4. Discussion

Here, the studies presented demonstrate that the natural flavonoid QUER is an effective inhibitor of in vitro JUNV infection, affecting the viral replication cycle in a dose-dependent manner. The main inhibitory action of QUER was detected by treatment of A549 and Vero cell cultures prior to viral infection. From data presented in Figure 1A and Figure 2B, the selectivity index (SI: ratio CC_50_/EC_50_) calculated for QUER against JUNV infection of Vero and A549 cells is in the order 30.6–36.8. Then, it can be concluded that the selective antiviral action of QUER is due to an effect of the drug on JUNV multiplication and not to a toxic effect on cells. The cytostatic effects on the growth of Vero and A549 cells exhibited by QUER (Figure 1B) did not dismiss this conclusion since the in vitro antiviral activity was studied in non-proliferative cells at QUER concentrations lacking cytotoxicity. The induction of an antiviral state into the host cell was more evident when pretreatment was carried out for short periods of time before virus infection, such as 2 and 6 h, but it decreased significantly when preincubation with QUER was extended to 24 h. Accordingly, it was described that intracellular levels of QUER or its metabolites peak at 5 h after cell exposition to the drug and slowly decline over time [32,33], exerting a reversible inhibitory effect on cell culture.

QUER also showed antiviral action against JUNV when it was added after virus infection, but with minor effectiveness. In fact, about 7–20-fold increases in EC_50_ values, depending on the host cell, were observed by post-treatment in comparison to treatment before infection. Additionally, QUER was able to inactivate JUNV infectivity but this virucidal activity was very weak and only detected at high concentrations (Figure 3). At a concentration of 50 µg/mL, only a 25% reduction in remaining virus infectivity was detected by direct incubation of JUNV particles with QUER, whereas the inhibition in virus production by 2–6 h of cell pretreatment was about 75–85%, indicating that the blockade in JUNV infection can be mainly ascribed to an interference with any other process during viral multiplication. Furthermore, virus inactivation was not effective when JUNV was incubated with QUER at 4° C, in accordance with the powerless virucidal activity detected at 37 °C. Only very potent viral inactivating agents that produce an important disassembly of the viral particle maintain their inhibitory activity at 4 °C, whereas weak virucidal compounds are only active at higher temperatures [34,35,36].

Consistent with findings for other viruses [5,37,38,39,40,41,42], the analysis of the possible target affected in the cycle of JUNV multiplication through a time of addition assay showed that the greatest reduction in virus yields were obtained when QUER was present during the early stages of virus replication. However, the addition of the compound at delayed time points resulted in a minor but significant inhibition, indicating a possible secondary effect on a late stage of the virus cycle.

The study of virus entry allowed us to conclude that QUER affected both processes of JUNV adsorption and internalization (Figure 4C,D). A significant reduction of infectivity was observed when the compound at a concentration of 50 µg/mL was present only either during adsorption (65% inhibition) or during internalization (50% inhibition). As above mentioned, the inhibitory effect of QUER on JUNV infection by cell treatment before infection is higher (85% inhibition), suggesting that the compound induces any alteration into the cell state that mainly affects virus entry. However, we cannot discard any minor effect on a later step of the virus cycle, as it is also detected in time of addition experiments (Figure 4A,B).

Since JUNV entry is dependent on the initial virion attachment to the cell receptor TfR1, we explored the possibility that a minor presence of this molecule on the cell membrane could be involved on the disturbance of JUNV infection induced by QUER. Flavonoids and their metabolites are reported to act on several signaling cascades, such as protein and lipid kinase pathways, and their inhibitory or stimulatory actions on these events strongly affect cell function by altering the phosphorylation state of target molecules and modulating gene expression. Particularly, PI3K/Akt is one of the major signaling pathways involved in cell survival, proliferation, migration and apoptosis and are shown to be widely involved in events related to multiplication of several viruses, including JUNV [24,43,44]. The PI3K/Akt pathway is responsible for iron homoeostasis because it is directly involved in the Tf and the TfRs recycling pathway. In all types of mammalian cells, iron is an essential trace element for cell growth and division, synthesis of DNA and diverse metabolic processes, in addition to being involved in the progression of oxidative stress [45]. Tf is an iron-binding protein that facilitates iron-uptake in cells, binds TfR at the membrane and enters the cell via clathrin-mediated endocytosis [46]. Tf remains associated with its receptor after endocytosis and dissociates only after recycling of the complex to the plasma membrane [46,47]. PI3K is required for membrane traffic, and its inhibitors like wortmannin, LY294002 and QUER reduced the rate of Tf-TfR recycling [47,48]. On this basis, and supported by our previous results showing that JUNV modulates the PI3K/Akt/PKB pathway to achieve an efficient replication process [24], the effects on Akt and TfR1 after short treatment of A549 cells with QUER were evaluated. A strong inhibition of Akt phosphorylation without affecting total protein expression was demonstrated, consistent with the widely reported fact that Akt is an important downstream effector of PI3K. This inhibition of Akt phosphorylation was reflected in a change in the fluorescence distribution of TfR1 in the treated cells, turning from a well-defined membrane pattern to a mainly cytoplasmic dotted one but, as evidenced by WB, the protein expression levels were not modified. The influence of QUER on the initial events of the viral multiplication cycle that comprise the entry process as a whole were also reported for other viruses; in these cases, the PI3K/Akt/PKB pathway would also be involved [13,39].

By contrast, low or undetectable levels of viral inhibition were detected with QUER cell treatment prior to infection with the arenaviruses TCRV and LCMV. The participation of PI3K in LCMV infection was described in two studies with contradictory results. Ref. [49] reported that treatment with the PI3K inhibitor wortmannin affected LCMV infection by blockade of virus entry into the cell. On the contrary, other research demonstrated that inhibition of PI3K/Akt with LY294002 affected viral budding and, to a minor degree, RNA synthesis, but virus entry was not impaired [50]. The divergent findings between these two reports, and also with our results, may be attributed to several differences in the experimental conditions, such as the PI3K inhibitor (abovementioned), virus strain and time of treatment. The publications about LCMV employed the Armstrong strain [50] and the cl-13, an Armstrong-derived isolate [49], whereas we have used the WE strain. It is well known the great variability registered in biological and immunological behavior among the diverse LCMV strains usually employed in research [51]. Furthermore, in both studies cells were always treated with the inhibitors after LCMV adsorption, in contrast with our schedule of cell pretreatment before viral infection. Although the reasons of these discrepancies are not easy to elucidate at the present, clearly, for several arenaviruses, there is a different usage of cell receptor for virus entry in comparison with JUNV: OW viruses, such as LASV and LCMV, as well as non-pathogenic viruses of clade B such as TCRV, AMAV and CUPV viruses that do not bind to human TfR1 [25,26,28,52,53]. Therefore, the data with TCRV and LCMV in Figure 5 support our proposal about the involvement of the abundance of TfR1 in the cell membrane, controlled by PI3K/Akt, in the inhibitory activity of QUER against JUNV infection.

In conclusion, QUER is an active inhibitor of JUNV by induction of a cellular antiviral state that mainly affects viral entry, probably modulated through the effect of the failure in AKT phosphorylation on TfR1 recycling to cell membrane. However, it must be noted that the antiproliferative effect of QUER is also mediated by the inhibition of the PI3K-Akt/PKB pathway [54], and both QUER and TfR1 are involved in multiple signaling pathways, such as IKK–NF-κB, MAPK/ERK MAPK/p38 or p53 [11,55,56,57]. Since several of these pathways are required for the multiplication of JUNV [58,59,60,61], it cannot be discarded that the inhibitory action of QUER on JUNV could be the resultant of different simultaneous effects.

## Data Availability

The data that support the findings of this study are available from the corresponding author upon reasonable request.

## References

[1] Linero F.N., Sepúlveda C.S., Giovannoni F., Castilla V., García C.C., Scolaro L.A., Damonte E.B. (2012). Host Cell Factors as Antiviral Targets in Arenavirus Infection. Viruses.

[2] Adesina A.S., Oyeyiola A., Obadare A., Igbokwe J., Abejegah C., Akhilomen P., Bangura U., Asogun D., Tobin E., Ayodeji O. (2023). Circulation of Lassa Virus across the Endemic Edo-Ondo Axis, Nigeria, with Cross-Species Transmission between Multimammate Mice. Emerg. Microbes Infect..

[3] McCormick J.B., King I.J., Webb P.A., Scribner C.L., Craven R.B., Johnson K.M., Elliott L.H., Belmont-Williams R. (1986). Lassa fever. Effective therapy with ribavirin. N. Engl. J. Med..

[4] Enria D.A., Briggiler A.M., Sánchez Z. (2008). Treatment of Argentine Hemorrhagic Fever. Antivir. Res..

[5] Shorobi F.M., Nisa F.Y., Saha S., Chowdhury M.A.H., Srisuphanunt M., Hossain K.H., Rahman M.A. (2023). Quercetin: A Functional Food-Flavonoid Incredibly Attenuates Emerging and Re-Emerging Viral Infections through Immunomodulatory Actions. Molecules.

[6] Peng D., Chen L., Sun Y., Sun L., Yin Q., Deng S., Niu L., Lou F., Wang Z., Xu Z. (2020). Melanoma Suppression by Quercein Is Correlated with RIG-I and Type I Interferon Signaling. Biomed. Pharmacother..

[7] Yuan Z., Long C., Junming T., Qihuan L., Youshun Z., Chan Z. (2012). Quercetin-Induced Apoptosis of HL-60 Cells by Reducing PI3K/Akt. Mol. Biol. Rep..

[8] Saeedi-Boroujeni A., Mahmoudian-Sani M.R. (2021). Anti-Inflammatory Potential of Quercetin in COVID-19 Treatment. J. Inflamm..

[9] Li Y., Yao J., Han C., Yang J., Chaudhry M.T., Wang S., Liu H., Yin Y. (2016). Quercetin, Inflammation and Immunity. Nutrients.

[10] Khan F., Niaz K., Maqbool F., Hassan F.I., Abdollahi M., Nagulapalli Venkata K.C., Nabavi S.M., Bishayee A. (2016). Molecular Targets Underlying the Anticancer Effects of Quercetin: An Update. Nutrients.

[11] Asgharian P., Tazekand A.P., Hosseini K., Forouhandeh H., Ghasemnejad T., Ranjbar M., Hasan M., Kumar M., Beirami S.M., Tarhriz V. (2022). Potential Mechanisms of Quercetin in Cancer Prevention: Focus on Cellular and Molecular Targets. Cancer Cell Int..

[12] Yang D., Wang T., Long M., Li P. (2020). Quercetin: Its Main Pharmacological Activity and Potential Application in Clinical Medicine. Oxid. Med. Cell. Longev..

[13] Di Petrillo A., Orrù G., Fais A., Fantini M.C. (2022). Quercetin and Its Derivates as Antiviral Potentials: A Comprehensive Review. Phytother. Res..

[14] Lalani S., Poh C.L. (2020). Flavonoids as Antiviral Agents for Enterovirus A71 (EV-A71). Viruses.

[15] Rojas N., Del Campo J.A., Clement S., Lemasson M., García-Valdecasas M., Gil-Gómez A., Ranchal I., Bartosch B., Bautista J.D., Rosenberg A.R. (2016). Effect of Quercetin on Hepatitis C Virus Life Cycle: From Viral to Host Targets. Sci. Rep..

[16] Zhao Y., Chen B., Shen J., Wan L., Zhu Y., Yi T., Xiao Z. (2017). The Beneficial Effects of Quercetin, Curcumin, and Resveratrol in Obesity. Oxid. Med. Cell. Longev..

[17] Wang G., Wang Y., Yao L., Gu W., Zhao S., Shen Z., Lin Z., Liu W., Yan T. (2022). Pharmacological Activity of Quercetin: An Updated Review. Evid.-Based Complement. Altern. Med..

[18] Neamtu A.A., Maghiar T.A., Alaya A., Olah N.K., Turcus V., Pelea D., Totolici B.D., Neamtu C., Maghiar A.M., Mathe E. (2022). A Comprehensive View on the Quercetin Impact on Colorectal Cancer. Molecules.

[19] Zarenezhad E., Abdulabbas H.T., Kareem A.S., Kouhpayeh S.A., Barbaresi S., Najafipour S., Mazarzaei A., Sotoudeh M., Ghasemian A. (2023). Protective Role of Flavonoids Quercetin and Silymarin in the Viral-Associated Inflammatory Bowel Disease: An Updated Review. Arch. Microbiol..

[20] Colunga Biancatelli R.M.L., Berrill M., Catravas J.D., Marik P.E. (2020). Quercetin and Vitamin C: An Experimental, Synergistic Therapy for the Prevention and Treatment of SARS-CoV-2 Related Disease (COVID-19). Front. Immunol..

[21] Ramos P.R.P.d.S., Mottin M., Lima C.S., Assis L.R., de Oliveira K.Z., Mesquita N.C.d.M.R., Cassani N.M., Santos I.A., Borba J.V.V.B., Fiaia Costa V.A. (2022). Natural Compounds as Non-Nucleoside Inhibitors of Zika Virus Polymerase through Integration of In Silico and In Vitro Approaches. Pharmaceuticals.

[22] Contigiani M.S., Sabattini M.S. (1977). Virulencia Diferencial de Cepas de Virus Junín Por Marcadores Biológicos En Ratones y Cobayos. Medicina.

[23] Mosmann T. (1983). Rapid Colorimetric Assay for Cellular Growth and Survival: Application to Proliferation and Cytotoxicity Assays. J. Immunol. Methods.

[24] Linero F.N., Scolaro L.A. (2009). Participation of the Phosphatidylinositol 3-Kinase/Akt Pathway in Junín Virus Replication in Vitro. Virus Res..

[25] Cao W., Henry M.D., Borrow P., Yamada H., Elder J.H., Ravkov E.V., Nichol S.T., Compans R.W., Campbell K.P., Oldstone M.B. (1998). Identification of alpha-dystroglycan as a receptor for lymphocytic choriomeningitis virus and Lassa fever virus. Science.

[26] Spiropoulou C.F., Kunz S., Rollin P.E., Campbell K.P., Oldstone M.B.A. (2002). New World Arenavirus Clade C, but Not Clade A and B Viruses, Utilizes α-Dystroglycan as Its Major Receptor. J. Virol..

[27] Radoshitzky S.R., Abraham J., Spiropoulou C.F., Kuhn J.H., Nguyen D., Li W., Nagel J., Schmidt P.J., Nunberg J.H., Andrews N.C. (2007). Transferrin Receptor 1 Is a Cellular Receptor for New World Haemorrhagic Fever Arenaviruses. Nature.

[28] Hallam S.J., Koma T., Maruyama J., Paessler S. (2018). Review of Mammarenavirus Biology and Replication. Front. Microbiol..

[29] Matter W.F., Brown R.F., Vlahosl C.J. (1992). The Inhibition of Phosphatidylinositol 3-Kinase by Quercetin and Analogs. Biochem. Biophys. Res. Commun..

[30] Walker E.H., Pacold M.E., Perisic O., Stephens L., Hawkins P.T., Wymann M.P., Williams R.L. (2000). Structural Determinants of Phosphoinositide 3-Kinase Inhibition by Wortmannin, LY294002, Quercetin, Myricetin, and Staurosporine. Mol. Cell.

[31] Riaz M.K., Zhang X., Wong K.H., Chen H., Liu Q., Chen X., Zhang G., Lu A., Yang Z. (2019). Pulmonary Delivery of Transferrin Receptors Targeting Peptide Surface-Functionalized Liposomes Augments the Chemotherapeutic Effect of Quercetin in Lung Cancer Therapy. Int. J. Nanomed..

[32] Kim M.K., Park K.S., Yeo W.S., Choo H., Chong Y. (2009). In Vitro Solubility, Stability and Permeability of Novel Quercetin-Amino Acid Conjugates. Bioorg. Med. Chem..

[33] Kim M.K., Park K.S., Lee C., Park H.R., Choo H., Chong Y. (2010). Enhanced Stability and Intracellular Accumulation of Quercetin by Protection of the Chemically or Metabolically Susceptible Hydroxyl Groups with a Pivaloxymethyl (POM) Promoiety. J. Med. Chem..

[34] Wurtz N., Hasni I., Bancod A., La Scola B. (2022). Confirmatory Virucidal Activity of Ionised Active Water S-100® on the SARS-CoV-2 Virus. Adv. Virol..

[35] Tang J., Colacino J.M., Larsen S.H., Spitzer W. (1990). Virucidal Activity of Hypericin against Enveloped and Non-Enveloped DNA and RNA Viruses. Antivir. Res..

[36] García C.C., Talarico L., Almeida N., Colombres S., Duschatzky C., Damonte E.B. (2003). Virucidal Activity of Essential Oils from Aromatic Plants of San Luis, Argentina. Phytother. Res..

[37] Li K., Zang X., Meng X., Li Y., Xie Y., Chen X. (2022). Targeted Delivery of Quercetin by Biotinylated Mixed Micelles for Non-Small Cell Lung Cancer Treatment. Drug Deliv..

[38] Lee G., Kang H.R., Kim A., Park J.H., Lee M.J., Kim S.M. (2023). Preventive Effects of Quercetin against Foot-and-Mouth Disease Virus in Vitro and in Vivo by Inducing Type I Interferon. Front. Microbiol..

[39] Ganesan S., Faris A.N., Comstock A.T., Wang Q., Nanua S., Hershenson M.B., Sajjan U.S. (2012). Quercetin Inhibits Rhinovirus Replication in Vitro and in Vivo. Antivir. Res..

[40] Lee S., Lee H.H., Shin Y.S., Kang H., Cho H. (2017). The Anti-HSV-1 Effect of Quercetin Is Dependent on the Suppression of TLR-3 in Raw 264.7 Cells. Arch. Pharm. Res..

[41] Mehrbod P., Abdalla M.A., Fotouhi F., Heidarzadeh M., Aro A.O., Eloff J.N., McGaw L.J., Fasina F.O. (2018). Immunomodulatory Properties of Quercetin-3-O-α-L-Rhamnopyranoside from Rapanea Melanophloeos against Influenza a Virus. BMC Complement. Altern. Med..

[42] Chiow K.H., Phoon M.C., Putti T., Tan B.K.H., Chow V.T. (2016). Evaluation of Antiviral Activities of Houttuynia Cordata Thunb. Extract, Quercetin, Quercetrin and Cinanserin on Murine Coronavirus and Dengue Virus Infection. Asian Pac. J. Trop. Med..

[43] Ji W.-T., Liu H.J. (2008). PI3K-Akt Signaling and Viral Infection. Recent Pat. Biotechnol..

[44] Cooray S. (2004). The Pivotal Role of Phosphatidylinositol 3-Kinase-Akt Signal Transduction in Virus Survival. J. Gen. Virol..

[45] Shen Y., Li X., Dong D., Zhang B., Xue Y., Shang P. (2018). Transferrin Receptor 1 in Cancer: A New Sight for Cancer Therapy. Am. J. Cancer Res..

[46] Mayle K.M., Le A.M., Kamei D.T. (2012). The Intracellular Trafficking Pathway of Transferrin. Biochim. Biophys. Acta Gen. Subj..

[47] Van Dam E.M., Ten Broeke T., Jansen K., Spijkers P., Stoorvogel W. (2002). Endocytosed Transferrin Receptors Recycle via Distinct Dynamin and Phosphatidylinositol 3-Kinase-Dependent Pathways. J. Biol. Chem..

[48] Matthew-Onabanjo A.N., Janusis J., Mercado-Matos J., Carlisle A.E., Kim D., Levine F., Cruz-Gordillo P., Richards R., Lee M.J., Shaw L.M. (2020). Beclin 1 Promotes Endosome Recruitment of Hepatocyte Growth Factor Tyrosine Kinase Substrate to Suppress Tumor Proliferation. Cancer Res..

[49] Pasqual G., Rojek J.M., Masin M., Chatton J.-Y., Kunz S. (2011). Old World Arenaviruses Enter the Host Cell via the Multivesicular Body and Depend on the Endosomal Sorting Complex Required for Transport. PLoS Pathog..

[50] Urata S., Ngo N., de la Torre J.C. (2012). The PI3K/Akt Pathway Contributes to Arenavirus Budding. J. Virol..

[51] Zhou X., Ramachandran S., Mann M., Popkin D.L. (2012). Role of Lymphocytic Choriomeningitis Virus (LCMV) in Understanding Viral Immunology: Past, Present and Future. Viruses.

[52] Sarute N., Ross S.R. (2017). New World Arenavirus Biology. Annu. Rev. Virol..

[53] Abraham J., Kwong J.A., Albarino C.G., Lu J.G., Radoshitzky S.R., Salazar-Bravo J., Farzan M., Spiropoulou C.F., Choe H. (2009). Host-Species Transferrin Receptor 1 Orthologs Are Cellular Receptors for Nonpathogenic New World Clade B Arenaviruses. PLoS Pathog..

[54] Gulati N., Laudet B., Zohrabian V.M., Murali R., Jhanwar-Uniyal M. (2006). The Antiproliferative Effect of Quercetin in Cancer Cells is Mediated via Inhibition of the PI3K-Akt/PKB Pathway. Anticancer Res..

[55] Kenneth N.S., Mudie S., Naron S., Rocha S. (2013). TfR1 Interacts with the IKK Complex and Is Involved in IKK-NF-ΚB Signalling. Biochem. J..

[56] Gangwar V., Garg A., Lomore K., Korla K., Bhat S.S., Rao R.P., Rafiq M., Kumawath R., Uddagiri B.V., Kareenhalli V.V. (2021). Immunomodulatory Effects of a Concoction of Natural Bioactive Compounds—Mechanistic Insights. Biomedicines.

[57] Campisi A., Bonfanti R., Raciti G., Bonaventura G., Legnani L., Magro G., Pennisi M., Russo G., Chiacchio M.A., Pappalardo F. (2020). Gene Silencing of Transferrin-1 Receptor as a Potential Therapeutic Target for Human Follicular and Anaplastic Thyroid Cancer. Mol. Ther. Oncolytics.

[58] Rodrigo W.W.S.I., Ortiz-Riaño E., Pythoud C., Kunz S., de la Torre J.C., Martínez-Sobrido L. (2012). Arenavirus Nucleoproteins Prevent Activation of Nuclear Factor Kappa B. J. Virol..

[59] Fitzpatrick C.J., Mudhasani R.R., Altamura L.A., Campbell C.E., Tran J.P., Beitzel B.F., Narayanan A., de la Fuente C.L., Kehn-Hall K., Smith J.M. (2022). Junin Virus Activates P38 MAPK and HSP27 Upon Entry. Front. Cell. Infect. Microbiol..

[60] Rodríguez M.E., Brunetti J.E., Wachsman M.B., Scolaro L.A., Castilla V. (2014). Raf/MEK/ERK Pathway Activation Is Required for Junín Virus Replication. J. Gen. Virol..

[61] Brunetti J.E., Quintana V.M., Scolaro L.A., Castilla V. (2022). Inhibitors of the P38 Cell Signaling Pathway as Antiviral Compounds against Junín Virus. Arch. Virol..

